# HPRT-DETR: A High-Precision Real-Time Object Detection Algorithm for Intelligent Driving Vehicles

**DOI:** 10.3390/s25061778

**Published:** 2025-03-13

**Authors:** Xiaona Song, Bin Fan, Haichao Liu, Lijun Wang, Jinxing Niu

**Affiliations:** School of Mechanical Engineering, North China University of Water Resources and Electric Power, Zhengzhou 450045, China; songxiaona1@126.com (X.S.); 201721819@stu.ncwu.edu.cn (B.F.); liuhaichao@ncwu.edu.cn (H.L.); niujinxing@ncwu.edu.cn (J.N.)

**Keywords:** intelligent driving vehicles, small target detection, RT-DETR, multi-scale fusion, deformable attention

## Abstract

Object detection is essential for the perception systems of intelligent driving vehicles. RT-DETR has emerged as a prominent model. However, its direct application in intelligent driving vehicles still faces issues with the misdetection of occluded or small targets. To address these challenges, we propose a High-Precision Real-Time object detection algorithm (HPRT-DETR). We designed a Basic-iRMB-CGA (BIC) Block for a backbone network that efficiently extracts features and reduces the model’s parameters. We thus propose a Deformable Attention-based Intra-scale Feature Interaction (DAIFI) module by combining the Deformable Attention mechanism with the Intra-Scale Feature Interaction module. This enables the model to capture rich semantic features and enhance object detection accuracy in occlusion. The Local Feature Extraction Fusion (LFEF) block was created by integrating the local feature extraction module with the CNN-based Cross-scale Feature Fusion (CCFF) module. This integration expands the model’s receptive field and enhances feature extraction without adding learnable parameters or complex computations, effectively minimizing missed detections of small targets. Experiments on the KITTI dataset show that, compared to RT-DETR, HPRT-DETR improves mAP50 and FPS by 1.98% and 15.25%, respectively. Additionally, its generalization ability is assessed on the SODA 10M dataset, where HPRT-DETR outperforms RT-DETR in most evaluation metrics, confirming the model’s effectiveness.

## 1. Introduction

At present, artificial intelligence technology is developing rapidly. As an important branch of this field, intelligent driving vehicles are constantly breaking through technical bottlenecks and promoting the innovation of transportation modes. Object detection serves as a fundamental component of environmental perception in intelligent driving vehicles. Its primary objective is to accurately and rapidly locate and identify various traffic participants within complex traffic scenarios, thereby ensuring the safe driving of intelligent driving vehicles. In real-world traffic driving scenes, most of the detection targets are in complex backgrounds, and the scales of the targets change significantly, with mutual occlusion among the targets. Many object detection models, such as the YOLO series and DETR series, have achieved good detection results. However, when these models are directly applied to the object detection of intelligent driving vehicles, challenges arise. Specifically, if the targets are small or obstructed by other objects, these models may still experience missed or false detection. Therefore, it is essential to develop a high-precision real-time object detection algorithm that is suitable for intelligent driving vehicles.

To address the challenges associated with object detection, several models based on Convolutional Neural Networks (CNNs) have been developed, including Faster R-CNN [1], Mask R-CNN [2], Cascade R-CNN [3], SSD [4], and the YOLO series. In 2017, Vaswani et al. proposed the Transformer model, which effectively addressed the long-distance dependency problem by capturing global contextual relationships [5]. The remarkable success of this model in the field of Natural Language Processing (NLP) has prompted an increasing number of researchers to focus their attention on the Transformer model. In 2020, Facebook AI launched the Detection Transformer (DETR) model, which was the first end-to-end object detection model using the Transformer structure [6]. It can directly predict the position and category of objects without complex post-processing. In 2023, the RT-DETR model developed by Zhao et al. demonstrated superior performance in both detection accuracy and speed compared to concurrent YOLO models [7]. This achievement was made possible through Attention-based Intra-scale Feature Interaction (AIFI) and CNN-based Cross-scale Feature Fusion (CCFF), as well as the comprehensive utilization of image feature extraction methods provided by the backbone network. Despite the remarkable performance of the RT-DETR model, it still encounters several challenges when applied to real-world traffic scenarios. These challenges include the issues of missed detections and false detections when confronted with small targets and occlusion situations. In light of this, we develop a high-precision real-time object detection model, namely HPRT-DETR. This model not only reduces the complexity and computational load but also extracts multi-scale semantic features. Consequently, it significantly decreases the rates of missed detection and false detection. Our main contributions are summarized as follows.

1. We propose an efficient and lightweight feature extraction module for the backbone network, namely the Basic-iRMB-CGA Block (BIC Block). The network architecture design of this module is inspired by the Inverted Residual Mobile Block (iRMB). By integrating convolution operations with a Cascaded Group Attention mechanism, the module can capture local features and global contextual information, thereby significantly enhancing the model’s feature representation capability. To effectively reduce the complexity of the model, this study also incorporates depthwise separable convolutions, enabling real-time inference capabilities while maintaining a high detection accuracy.
2. We developed the Deformable Attention-based Intra-scale Feature Interaction (DAIFI) module, which is designed to effectively capture essential features. The Multi-head Attention in the AIFI module of RT-DETR is replaced by Deformable Attention, forming the DAIFI module. This module enhances the model’s capability to capture essential features and concentrate on critical areas in the image. As a result, it can accurately identify targets even in situations where they may be occluded.
3. We employed Local Feature Extraction to enhance the Fusion Block module within the CNN-based Cross-scale Feature Fusion module, thereby constructing a Local Feature Extraction Fusion (LFEF) Block that efficiently extracts local features. The shift-convolution operation in this module can provide a larger receptive field than the 1 × 1 convolution without introducing additional learnable parameters or computational burden. As a result, it can extract the local features of images efficiently and reduce the missed detection rate of small objects.

The remainder of this paper is organized as follows. Section 2 outlines the current status and limitations of object detection, as well as the primary motivations behind the proposed algorithm. The model architecture of the HPRT-DETR is detailed in Section 3. In Section 4, we comprehensively evaluate the effectiveness of the proposed algorithm. Finally, Section 5 summarizes the findings of this study and offers insights into ongoing challenges and future research trends in this field.

## 2. Related Work

With the advancement of the deep learning theory, numerous distinct types of models have emerged in the domain of object detection. The R-CNN series, ranging from the R-CNN [8] to Mask R-CNN, has been constantly improving the approaches for generating candidate regions and extracting features, thereby enhancing the detection performance. The YOLO series, ranging from v1 to v10, is known for its efficiency of single-stage detection. The network architecture and detection mechanism are continuously refined, achieving an optimal balance between real-time performance and accuracy. SSD conducts detection by setting default boxes on multi-scale feature maps, yielding good detection effects for small objects and fast processing speed. DETR employs the Transformer architecture, simplifying the detection procedure. The CenterNet [9] series converts detection into a keypoint detection issue and CenterNet++ further improves performance, achieving high detection precision and efficiency. Swin-Transformer [10], which is based on the Transformer architecture, achieves high-accuracy detection through set prediction. EfficientDet [11] ensures high precision while facilitating real-time detection by employing an efficient backbone network and a well-designed detection head. These models have unique features and advantages in various applications and datasets, driving the ongoing progress of object detection.

In real-world traffic scenarios, intelligent driving object detection faces numerous challenges, including mutual occlusion among detection targets, variations in the scale of traffic targets, and complex background environments. Numerous researchers have developed relevant algorithms to address these challenges. In the study conducted by Li et al. [12], the traditional upsampling layer was replaced with a content-aware feature reconstruction module to enhance the fusion of image features. Additionally, the conventional convolutional structure was substituted with the SPD-Conv module, thereby improving the detection accuracy for low-resolution images and extremely small objects. Wang et al. [13] developed a multi-channel attention block to integrate features from various levels, thereby enhancing the foundational information for feature fusion within the model. They introduced an α factor based on Intersection over Union (IoU) [14] to improve the regression accuracy of object detection. Zhang et al. [15] integrated the Receptive Field Block (RFB) [16] module into the feature fusion to enhance feature diversity and reduce computational costs, thereby improving the network’s detection efficiency for multi-scale traffic targets. Tang et al. [17] enhanced the feature fusion module of YOLO by introducing a Dual Fusion Gradual Pyramid structure (DFGPN). This approach effectively addresses the feature attenuation in complex traffic scenarios. Additionally, target information is reinforced through a feature channel weighting strategy and the Residual Multi-head self-Attention (RMA) module, thereby reducing background interference. Qiu et al. [18] proposed the IDOD-YOLOv7 framework, which enhances the Image-Dehazing Object Detection (IDOD) module by utilizing parallel convolution to compensate for information loss during the convolution process, thereby improving image defogging quality. Additionally, the Self-Adaption Image Processing (SAIP) is introduced to ensure that the images meet the requirements for object detection. Khan et al. [19] enhanced the Localized Semantic Feature Mixers (LSFM) [20] model by advancing it from single pedestrian detection to multi-class traffic object detection, meeting the needs for multi-object detection in complex autonomous driving environments. Furthermore, several hyperspectral target detection methods based on the Transformer architecture have provided novel approaches for addressing the issues explored in this study [21,22,23].

Although the aforementioned algorithms have demonstrated satisfactory detection performance, there are still certain limitations that need to be addressed. DETR demonstrates superior performance in complex scenarios due to its end-to-end training method and global context perception ability, but it also has significant limitations. For instance, it suffers from a slow convergence speed during training, requires a substantial amount of training time, and displays suboptimal detection performance for small targets. These limitations restrict its application in scenarios that demand higher real-time performance. Subsequently, RT-DETR, an enhanced version of DETR, eliminated the Non-Maximum Suppression (NMS) step, thereby achieving true real-time performance. The comprehensive structural optimization of RT-DETR significantly improved its inference speed while maintaining high detection accuracy. Therefore, we selected RT-DETR as the benchmark model for the study. Although RT-DETR has achieved remarkable results in the field of object detection, it still faces some urgent problems to be solved in real-world traffic driving scenarios. Firstly, RT-DETR utilizes on ResNet18 or ResNet50 as its backbone network for feature extraction, which limits its feature extraction capabilities. The model parameters of this backbone network are relatively large, which adversely affects the speed of object detection. Moreover, the AIFI module in RT-DETR still employs a traditional Multi-head attention mechanism. This approach demonstrates insufficient capability in capturing critical feature information when processing high-level feature layers, making it challenging to fully extract deep semantic information from these feature layers. In addition, within the CCFF module, shallow features contribute little to detection performance and may even introduce noise. This can lead to a low efficiency in merging information from different scale features, thereby hindering the advantages offered by the CCFF module. The aforementioned issues may result in RT-DETR experiencing missed detection or false detection, particularly in scenarios involving small objects and occlusions.

Based on the analysis above, our improvement strategies are as follows. Firstly, a lightweight feature extraction module BIC Block is employed to address the issues of limited feature extraction capability and excessive computational burden. Furthermore, a Deformable Attention mechanism is introduced in the high-level feature extraction layer to construct an efficient feature extraction module, namely the DAIFI module. This approach aims to address the inadequate capability of capturing critical feature information within the high-level feature layers. Finally, the feature fusion module with an extensive receptive field, termed the LFEF Block, is integrated into the CNN-based Cross-scale Feature Fusion network. This integration addresses the issue of inefficient feature information fusion between multi-scale features.

## 3. Methods

### 3.1. The HPRT-DETR Model Architecture

**Figure 1:**
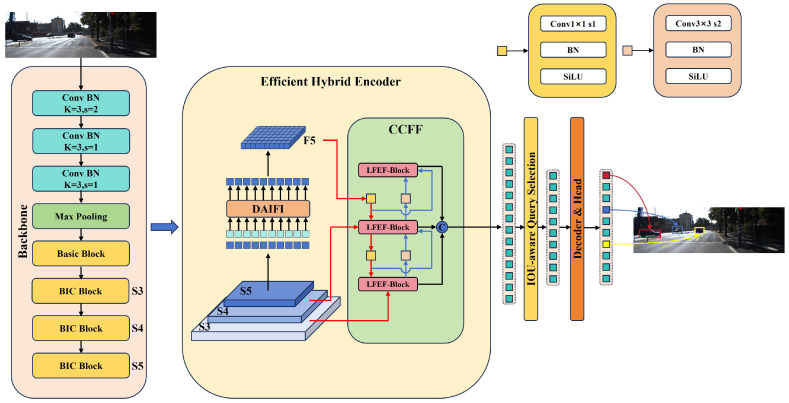
HPRT-DETR model structure diagram.

This paper proposes an efficient and lightweight HPRT-DETR model, the structure of which is shown in Figure 1. The model consists of four components: a backbone, an efficient hybrid encoder, a transformer decoder, and an auxiliary prediction head.

Firstly, the input image is fed into the backbone network, where a lightweight feature extraction module known as the BIC Block is utilized to extract multi-scale features S3, S4, and S5. Secondly, the features from S5 are fed into the DAIFI module to extract essential characteristics for generating F5, which is then reshaped to match the dimensions of S5. Subsequently, the S3, S4, and F5 features are fed into the CCFF module, where the LFEF Block module is employed to achieve efficient fusion of cross-scale features. Afterwards, a fixed number of image features are selected as initial object queries from the sequence of image features generated by the Encoder through IoU-aware Query Selection. Then, these initial object queries are fed into the Transformer Decoder. Finally, the decoder iteratively optimizes the initial object queries through auxiliary prediction heads, thereby generating precise bounding boxes and reliable confidence scores.

### 3.2. The Backbone Network Feature Extraction Module BIC Block

**Figure 2:**
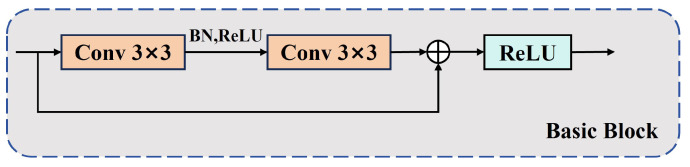
Basic Block structure diagram.

The RT-DETR model utilizes ResNet [24], which is based on a traditional CNN architecture, as the backbone network for feature extraction. Considering the trade-off between computational resource efficiency and model performance, this study selects the lightweight ResNet-18 as the foundational backbone network. ResNet-18 is mainly composed of multiple stacked Basic Block, the structure of which is shown in Figure 2.

**Figure 3:**
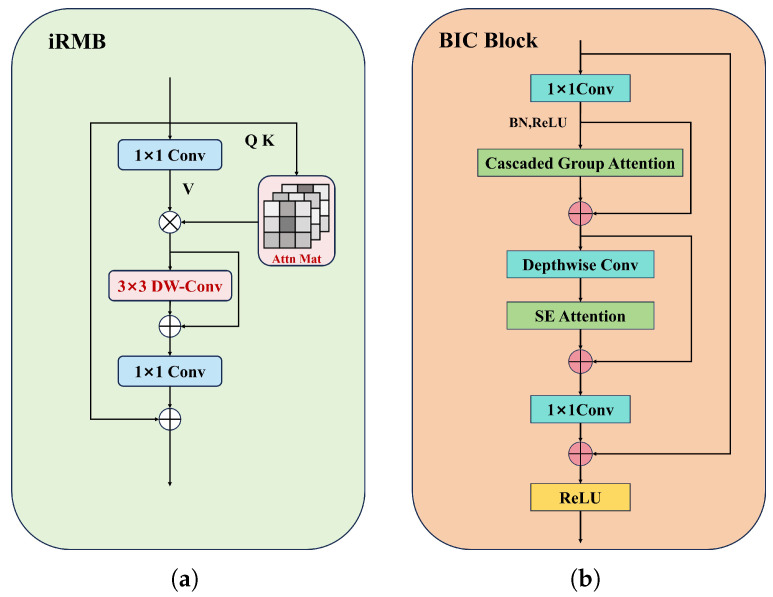
Inverted Residual Mobile Block (iRMB) and Basic-iRMB-CGA (BIC) Block structure diagram. (**a**) The iRMB structure diagram; (**b**) the BIC Block structure diagram.

The BIC Block primarily focuses on feature extraction within the confines of local receptive fields during the feature extraction process. This limitation hinders its ability to establish global dependencies between cross-regional feature information. To address the aforementioned issues, we design an efficient and lightweight backbone network feature extraction module, namely the BIC Block (as shown in Figure 3b). It integrates convolution operations with a Cascaded Group Attention mechanism [25], facilitating the collaborative extraction of local features and global features. The design inspiration for the BIC Block is derived from the iRMB network architecture [26] (as shown in Figure 3a).

The process of the BIC Block feature extraction is as follows. Firstly, 1 × 1 convolution is employed to increase the dimensionality of the input image features, thereby enhancing their expressive capability. Then, these features are subjected to Batch Normalization and ReLU activation before being fed into the Cascaded Group Attention for deep feature extraction. Specifically, this attention mechanism divides the input features into non-overlapping sub-features, with each sub-feature being assigned a corresponding attention head to process the feature information. The proposed method is capable of constructing global dependency relationships between cross-regional feature information more efficiently. However, this mechanism necessitates the computation of correlations between each position in the input sequence and all other positions during the feature extraction process. This results in a computational complexity that grows exponentially with the length of the input sequence. To address the aforementioned issues, this study utilizes depthwise separable convolution [27] to reduce the model’s computational parameters and enhance its computational efficiency. Finally, the output of the extracted image features is obtained through the application of the ReLU activation function.

The depthwise separable convolution is composed of depthwise convolution and 1 × 1 pointwise convolution. The ratio of parameter calculations between depthwise separable convolution and standard convolution is illustrated in Equation (1).

(1) $$\frac{Depthwise\mathrm{separable}\mathrm{convolution}}{Standard\mathrm{convolution}}=\frac{K\times K\times C_{in}+C_{in}\times C_{out}}{K\times K\times C_{in}\times C_{out}}=\frac{1}{C_{out}}+\frac{1}{K^{2}}$$

where K represents the size of the convolution kernel, and $C_{\mathrm{in}}$ and $C_{\mathrm{out}}$ represent the number of input and output channels, respectively. According to Equation (1), the incorporation of depthwise separable convolutions can significantly reduce the computational parameters of the model.

It is noteworthy that, in order to address the loss of inter-channel feature dependencies caused by the reduction of computational parameters in depthwise convolution, this study incorporates an SE attention [28] layer into the intermediate stage of depthwise separable convolutions. This approach enhances the model’s ability to comprehend the interdependencies among different channels by generating channel attention weights. At the same time, the BIC Block effectively alleviates the issue of vanishing gradients during the training process through the use of residual connections.

### 3.3. The DAIFI Module

**Figure 4:**
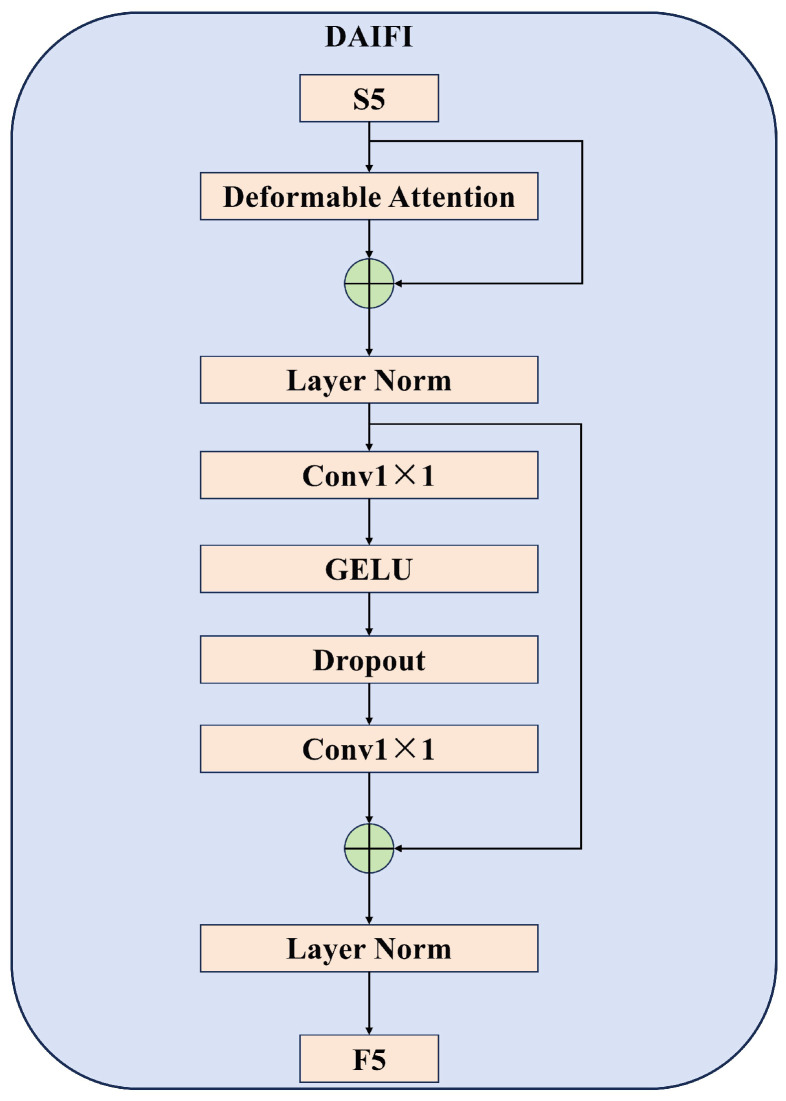
Deformable Attention-based Intra-scale Feature Interaction (DAIFI) module structure diagram.

The AIFI module in the RT-DETR model employs a single-layer Multi-head Attention Transformer encoder to process deep features. This design exhibits significant limitations when addressing complex scenarios. Specifically, in scenarios where occluded targets are present, traditional Multi-head Attention mechanisms struggle to effectively focus on the local features of the occluded regions due to their reliance on a uniform global attention weight allocation strategy. This limitation adversely affects the model’s detection accuracy. To address the aforementioned issues, this study integrates a Deformable Attention mechanism [29] into the intra-scale feature interaction module, replacing the existing Multi-head Attention mechanism and thereby constructing the DAIFI module. The structure of this module is illustrated in Figure 4. Compared to the original AIFI module, the DAIFI module enhances the model’s ability to focus on the feature information of key target areas by incorporating a Deformable Attention mechanism.

**Figure 5:**
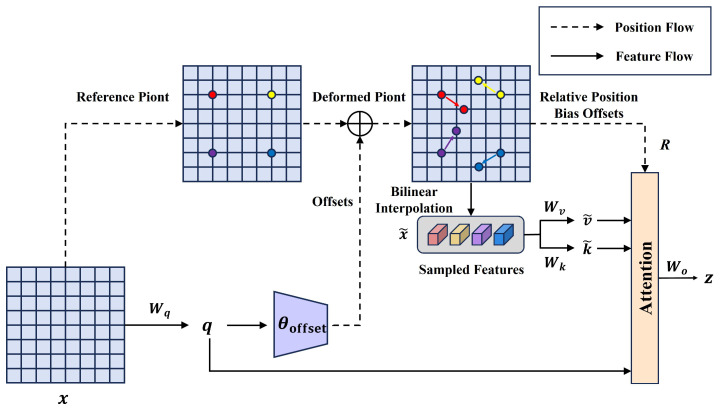
Deformable Attention mechanism structure diagram.

As illustrated in Figure 5, the core idea of the Deformable Attention mechanism is to introduce a learnable offset network that dynamically adjusts the spatial distribution of feature sampling points. This approach enables adaptive focusing on key regions within the input feature map. Specifically, this mechanism generates uniformly distributed reference points p based on the geometric characteristics of the input feature map. Simultaneously, it utilizes query features as input to predict spatial offsets Δp through an offset network, enabling the reference points to adaptively move towards key feature regions. Subsequently, the features of the deformed sampled points are extracted through bilinear interpolation. These features serve as the $\tilde{k}$ and $\tilde{v}$ after deformation, which will be utilized in subsequent attention calculations. Furthermore, this mechanism captures the spatial dependencies between features by calculating relative positional offsets through interpolation operations. Finally, the deformable attention weights z are computed by integrating the query features, the deformed key and value pairs, and the relative positional offsets. The final output features are obtained by performing a weighted sum of different attention weights, followed by a linear transformation. The detailed calculation process is as follows:

(2) $$\Delta p=\theta_{\mathrm{offset}}\left(q\right),\tilde{x}=\phi \left(x;p+\Delta p\right)$$

(3) $$q=xW_{q},\tilde{k}=\tilde{x}W_{k},\tilde{v}=\tilde{x}W_{v}$$

(4) $$z^{\left(m\right)}=\sigma \left(q^{\left(m\right)}{\tilde{k}}^{(m)⊤}/\sqrt{d}+\phi \left(\hat{B};R\right)\right){\tilde{v}}^{\left(m\right)}$$

(5) $$z=Concat\left(z^{\left(1\right)},z^{\left(2\right)},\ldots ,z^{\left(m\right)}\right)W_{o}$$

where ϕ(;) denotes the computations involved in bilinear interpolation, $W_{q},W_{k},W_{v}$, and $W_{o}$ represent the linear projection matrices for queries, keys, values, and output, m denotes the m-th head of attention, d represents the dimensionality of each head, and $\phi (\hat{B};R)$ signifies the relative position bias.

As illustrated in Figure 4, the output features processed by the Deformable Attention mechanism must sequentially undergo layer normalization, 1 × 1 convolution, and the GELU activation function for feature reorganization. This process provides dimensionally matched and reasonably distributed feature representations, facilitating subsequent cross-scale feature fusion.

### 3.4. LFEF Block

**Figure 6:**
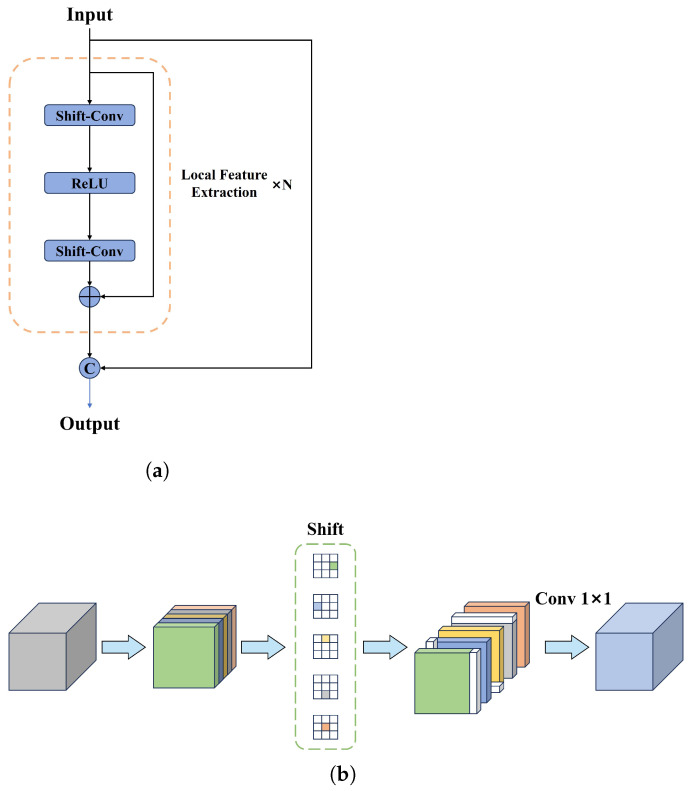
Local Feature Extraction Fusion (LFEF) Block and Shift-convolution structure diagram. (**a**) the LFEF Block structure diagram; (**b**) the Shift-convolution structure diagram.

In the CCFF module of the RT-DETR model, the Fusion Block processes features through two consecutive 1 × 1 convolutions. This design restricts its receptive field to 1 × 1. It results in a relatively limited contribution of shallow features to the overall detection performance. In some cases, shallow features can introduce noise, leading to missed detection of small targets. If we can efficiently fuse cross-scale features in images while simultaneously expanding the model’s receptive field, it will enhance the detection efficiency of small targets. Therefore, we utilize a Local Feature Extraction module (LFE) [30] to improve the Fusion Block within CCFF, thereby constructing a novel feature fusion module known as the LFEF Block. The LFEF block structure is illustrated in Figure 6a.

In Figure 6a, the LFEF Block consists of N groups of LFE modules. The final output is composed of the features obtained from the input characteristics, which are concatenated with the features extracted by N groups of local feature extraction modules through residual connections. The local feature extraction module contains shift-convolution. In the shift-convolution [31], we divide the input features into five equal groups. As shown in Figure 6b, the first four groups of features are shifted along different spatial dimensions (specifically left, right, up, and down), while the final group remains unchanged. This approach effectively leverages information from neighboring pixels. Without introducing additional learnable parameters and extensive computations, shift-convolution can offer a larger receptive field while maintaining computational complexity that is nearly identical to that of the original module. As a result, the LFEF Block can extract local features of images efficiently and reduce the missed detection rate of small objects.

## 4. Experiment

### 4.1. Datasets and Experimental Settings

In this study, the 2D object dataset from KITTI [32] is utilized to validate the efficiency of the proposed model. This dataset is one of the most commonly used evaluation datasets for computer vision algorithms in autonomous driving scenarios. It contains 7481 images collected from real-world traffic driving scenarios in urban areas, rural regions, and highways. The dataset comprises six categories of detection targets, including automobiles, trucks, vans, pedestrians, bicycles, and trams. In order to meet the requirements of the experiment, the dataset was segmented into training, validation, and testing subsets in a ratio of 7:2:1. At the same time, this study also employs the SODA 10M [33] dataset to evaluate the generalization capabilities of the model. This dataset comprises a total of 10,000 images. It includes six categories of detection targets: pedestrians, cars, trucks, trams, bicycles, and tricycles. The division ratio of this dataset is consistent with that of the KITTI dataset.

The experimental environment is based on the Windows operating system, Python 3.8.0, and PyTorch1.13.1. The hardware and model parameters are detailed in Table 1. The batch size was set to 4, with a training duration of 200 epochs, and the learning rate was set at 0.0001.

### 4.2. The Performance Comparison of Different Detection Models

**Figure 7:**
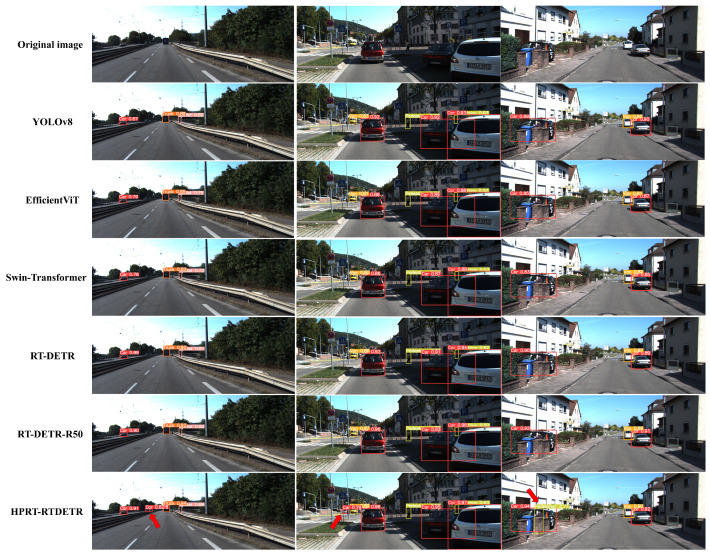
Comparison of detection results from different models.

To demonstrate the performance of the improved model in real-world traffic scenarios, we compared the proposed HPRT-DETR model with mainstream detection models including YOLOv8 [34], EfficientViT, Swin-Transformer, and the RT-DETR series. The comparison results are illustrated in Figure 7. The final row of Figure 7 presents the results of the HPRT-DETR model, achieving higher scores in multiple object detection categories than other models. The HPRT-DETR model demonstrates its capability to successfully identify small targets at long distances, vehicles obscured by traffic signs, and pedestrians obscured by walls (as indicated by the red arrows in the last row of Figure 7).

The results show that the HPRT-DETR model exhibits superior performance in addressing the challenges of small targets and obscured targets in real-world traffic scenarios. The reason for this is that the proposed DAIFI module and the LFEF Block module are capable of extracting high-level semantic features as well as multi-scale features.

To further highlight the advantages of the HPRT-DETR model, a comprehensive comparison of its performance metrics against those of other object detection models is conducted. The comparison results are presented in Table 2.

As shown in Table 2, the HPRT-DETR model has achieved significant improvements in both mAP50 and mAP50:95. The improvement in detection accuracy can be primarily attributed to the introduction of the DAIFI module and the LFEF Block. The DAIFI module is capable of more efficiently extracting semantic feature information from deep feature layers, thereby enhancing the detection accuracy of the model. The LFEF Block enhances the efficiency of cross-scale feature fusion by expanding the model’s receptive field, thereby improving the model’s capability to detect small targets. Moreover, as indicated in the last column of Table 2, the FPS performance of the HPRT-DETR model surpasses that of other models. This suggests that the model we proposed possesses a high detection speed. This is primarily attributed to the BIC Block. The introduction of BIC Block significantly reduces the number of model parameters, thereby alleviating the computational burden. It significantly improves the operational speed of the model while maintaining detection accuracy.

In summary, the HPRT-DETR model demonstrates superior performance in both detection accuracy and real-time. The improved model can meet the performance requirements of object detection algorithms in real-world traffic driving environments.

### 4.3. Experimental Details and Discussions

#### 4.3.1. Comparative Experiment of the Backbone Network

In real-world traffic driving environments, object detection algorithms must not only accurately locate pedestrians and vehicles but also possess excellent real-time performance. To enhance detection speed while maintaining detection accuracy, we optimized the backbone network of RT-DETR and introduced the BIC Block to reduce model parameters and computational load. We assessed the performance of the BIC Block by comparing it against mainstream backbone networks. These backbone networks primarily include the Basic Block [7], Star Block [35], WTConv Block [36], DualConv Block [37], AKConv Block [38], and DCNv2 Block [39]. This study utilizes the Basic Block derived from the backbone network ResNet-18 in RT-DETR. The comparative results are presented in Table 3.

As shown in the first row of Table 3, the Basic Block achieves performance metrics of 81.58% for precision, 76.59% for recall, 78.87% for mAP50, 49.69% for mAP50:95, 19.97 M for parameters, and 57.3 for GFLOPs. The detection performance exhibits a relatively balanced level.The Star-Block and WTConv-Block exhibit remarkable performance in reducing model parameters and computational load. However, their mAP50 values decrease by 2.43% and 1.22%, respectively. This indicates that, although these models are lightweight, their detection accuracies may be somewhat compromised. The performance of the DualConv-Block significantly lags behind that of the Basic Block, failing to achieve superior results in all evaluated metrics. While the AKConv-Block achieves a significant reduction in model parameters and computational load, it also experiences a decrease in mAP50. This backbone network is unable to maintain stable detection performance while reducing the consumption of computing resources. The DCNv2 Block demonstrates a substantial improvement in precision, achieving a 5.31% increase compared to the Basic Block. However, this enhancement is accompanied by an increase in model parameters and computational requirements, which may limit its applicability in resource-constrained environments. As shown in the last row of Table 3, the proposed BIC Block not only achieves substantial reductions in both the overall parameter and computational load but also enhances accuracy. This indicates that the BIC Block achieves a relatively optimal balance between model optimization and performance enhancement.

In conclusion, the comparative experimental results presented in Table 3 clearly demonstrate the excellent performance of the BIC Block across various performance metrics, thereby validating its effectiveness comprehensively.

#### 4.3.2. Experiment for Verifying the Effectiveness of the DAIFI Module

**Figure 8:**
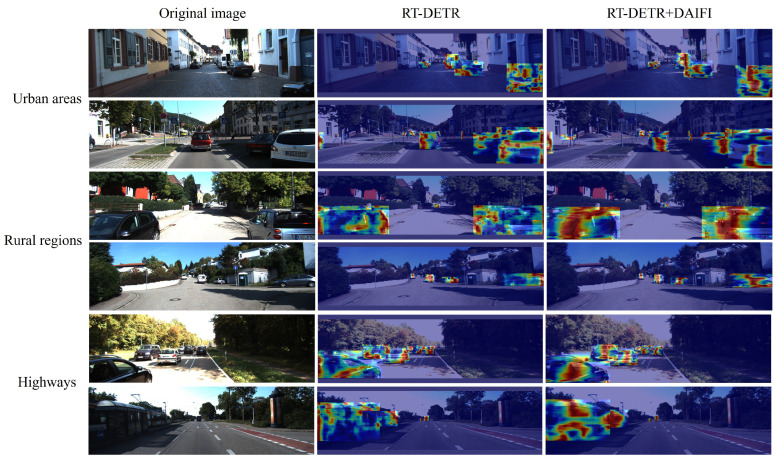
Comparison of model heatmaps before and after the introduction of the DAIFI module.

To validate the efficiency of the DAIFI module, we utilized GradCAM [40] to generate heatmaps both before and after the introduction of the Deformable Attention mechanism. Comparative experiments were carried out in various driving environments, including urban areas, rural regions, and highways. The comparative results of the heatmap are illustrated in Figure 8.

As shown in the second column of Figure 8, the RT-DETR model without the DAIFI module, the red areas in the heatmap are relatively dispersed. This indicates that the model is unable to focus on key areas effectively. Compared to the heatmap in the second column, the heatmap in the third column exhibits a more concentrated red region around the detected target and is positioned closer to the center of that target. The introduction of the DA-AIFI module enhances the precision and focus of the model’s attention. This allows the model to more effectively concentrate on key detection targets within images, thereby enabling accurate localization of target positions even in complex and variable backgrounds. The results presented in these heatmaps clearly illustrate the superior performance of the DAIFI module. This advantage is attributed to the module’s capability to precisely and flexibly extract high-level semantic features while capturing intricate details within images.

#### 4.3.3. The Ablation Experiments of Different Modules

To investigate the enhancement effects of the BIC Block, DAIFI module, and LFEF Block on the HPRT-DETR model, we conducted six sets of ablation studies. The experimental results are presented in Table 4.

From the data in Method 2 of Table 4, introducing the BIC Block module improves mAP50 and mAP50:95 by 0.65% and 0.45%, respectively, while reducing the parameter count and GFLOPs by 23.08% and 17.63%. This indicates that the model exhibits superior feature extraction capabilities and achieves faster detection speeds. From the data presented in Method 3 of Table 4, it is evident that integrating the DAIFI module into the model results in the increase of mAP50 by 0.34% and MAP50:95 by 0.11%, respectively, along with a small improvement in FPS. From the data in Method 4 of Table 4, the application of the LFEF Block leads to improvements in mAP50 and mAP50:95 by 0.73% and 0.56%, respectively, while also resulting in a slight increase in FPS. It has been demonstrated that the LFEF Block achieves a superior feature fusion effect. Finally, after incorporating all the improvement strategies, the HPRT-DETR model achieved increases of 1.98%, 2.03%, and 15.25% in mAP50, mAP50:95, and FPS, respectively. The ablation experiments demonstrate that each of the proposed improvement strategies contributes to the detection performance of the HPRT-DETR model.

#### 4.3.4. Model Generalization Ability Test

To further validate the efficiency of the models, we evaluated the generalization capabilities of both the RT-DETR and HPRT-DETR models using the SODA10M dataset. The results of the two models on the SODA10M dataset are presented in Table 5. As shown in Table 5, the performance metrics of HPRT-DETR consistently surpass those of RT-DETR.

**Figure 9:**
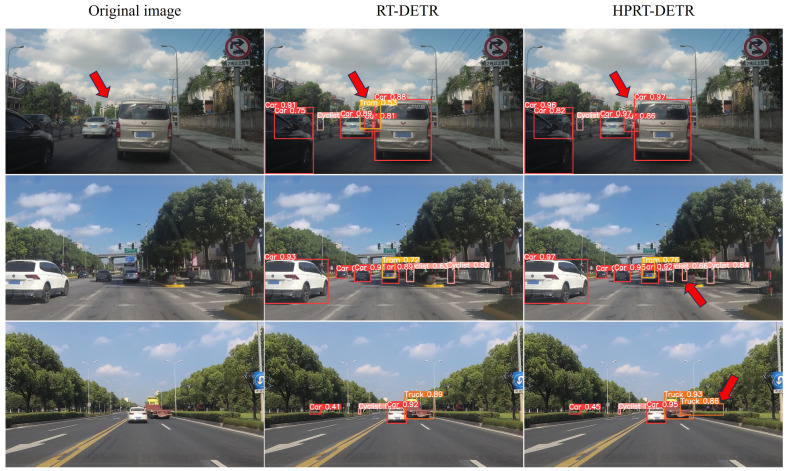
The visualization results of the RT-DETR and HPRT-DETR models on SODA 10M.

The visualization detection results of the RT-DETR and HPRT-DETR models on the SODA10M dataset are presented in Figure 9.

From the first row of Figure 9, it is evident that the RT-DETR model mistakenly identifies the background as the target Tram, while the HPRT-DETR model avoids this misdetection. From the second and third images in the last column (the areas highlighted by the red arrows), the HPRT-DETR model successfully detects a cyclist and a truck, which are not detected by the original RT-DETR model. The experimental data on generalization capability and the comparison results of visualization detection indicate that the HPRT-DETR model demonstrates superior detection performance and accuracy on the SODA10M dataset. Furthermore, the improved model is able to significantly reduce false and missed detection.

## 5. Conclusions

This study proposes a high-precision, real-time object detection model known as HPRT-DETR, which is specifically designed for intelligent driving vehicles. The model effectively reduces missed and false detection for small targets and occlusions, improving detection accuracy. It also enhances efficiency to meet the real-time requirements of intelligent vehicle object detection. HPRT-DETR features three key enhancements: the BIC Block that efficiently extract feature and effectively reduces the model’s parameters, the DAIFI module that captures essential features and lowers missed and false detection rates in occluded scenarios, and the LFEF Block for feature fusion that addresses missed detection of small targets. The superiority of the HPRT-DETR model’s performance is validated on the KITTI dataset and the SODA10M dataset. The HPRT-DETR model shows superior detection accuracy and speed, but it needs further improvement in traffic scenarios under complex weather conditions. In future work, we aim to address these issues to improve the performance of the model.

## Figures and Tables

**Table 1:** Hardware configuration and model parameters.

Types	Configuration	Types	Value
GPU	RTX A5000	Learning rate	0.0001
CPU	Intel i7-12700	Momentum	0.9
CUDA	11.7	Optimizer	AdamW
GPU Memory Size	24 G	Batch	4

**Table 2:** Comparison of evaluation metrics for different models.

Model	Precision (%)	Recall (%)	mAP50 (%)	mAP50:95 (%)	FPS (f/s)
YOLOv8	85.87	70.83	92.56	71.63	98
Efficient ViT	84.36	73.49	89.88	68.34	37
Swin-Transformer	83.19	72.71	88.64	67.57	19
RT-DETR	81.23	76.50	93.58	72.76	118
RT-DETR-R50	88.98	76.28	94.03	73.12	108
HPRT-DETR	89.67	76.15	95.56	74.79	136

**Table 3:** Comparison of evaluation metrics for different backbone networks.

Model	Precision (%)	Recall (%)	mAP50 (%)	mAP50:95 (%)	Params (M)	GFLOPs (G)
Basic Block	81.58	76.59	78.87	46.69	19.97	57.3
Star Block	78.56	74.81	76.44	48.83	18.76	53.2
WTConvBlock	86.66	71.68	77.65	49.34	17.56	51.7
DualConvBlock	76.80	74.41	76.14	48.78	20.86	59.8
AKConvBlock	83.26	75.64	77.89	48.98	16.55	50.4
DCNv2 Block	86.89	74.79	77.86	49.18	20.41	57.8
BIC Block	85.76	76.53	79.68	49.86	16.26	46.5

**Table 4:** Results of ablation experiments.

Method	BIC Block	DAIFI	LFEF Block	mAP50 (%)	mAP50:95 (%)	Params (M)	GFLOPs (G)	FPS (f/s)
1 (Basic)				93.58	72.76	19.97	57.3	118
2	🗸			94.23	73.21	15.36	47.2	149
3		🗸		93.92	72.87	19.85	56.5	122
4			🗸	94.31	73.32	19.97	57.5	120
5	🗸	🗸		94.98	73.55	15.26	46.2	141
6 (Ours)	🗸	🗸	🗸	95.56	74.79	15.19	46.1	136

**Table 5:** The results of the generalization ability of the model on the SODA 10M dataset.

Model	Precision (%)	Recall (%)	mAP50 (%)	mAP50:95 (%)	FPS (f/s)
RT-DETR	85.72	76.09	84.63	63.26	116
HPRT-DETR	88.63	76.36	86.28	70.54	128

## Data Availability

Data are contained within the article.
